# Sequential Plasma Metabolome and Proteome Analyses to Develop a Novel Monitoring Strategy for Patients with Epithelial Ovarian Cancer: A Pilot Study

**DOI:** 10.3390/ijms26125435

**Published:** 2025-06-06

**Authors:** Eiji Hishinuma, Shogo Shigeta, Naomi Matsukawa, Yasunobu Okamura, Ikuko N. Motoike, Takamichi Minato, Yusuke Shibuya, Jun Yasuda, Kengo Kinoshita, Seizo Koshiba, Muneaki Shimada

**Affiliations:** 1 Advanced Research Center for Innovations in Next-Generation Medicine, Tohoku University, Sendai 980-8573, Japan; eiji.hishinuma.e7@tohoku.ac.jp (E.H.);; 2 Tohoku Medical Megabank Organization, Tohoku University, Sendai 980-8573, Japan; 3 Department of Gynecology, Graduate School of Medicine, Tohoku University, Sendai 980-8574, Japan; shogo.shigeta.a4@tohoku.ac.jp (S.S.);; 4 Systems Bioinformatics, Graduate School of Information Sciences, Tohoku University, Sendai 980-8579, Japan; 5 Department of Obstetrics and Gynecology, Hachinohe City Hospital, Hachinohe 031-8555, Japan; 6 Division of Molecular and Cellular Oncology, Miyagi Cancer Center, Natori 981-1293, Japan

**Keywords:** biomarker, epithelial ovarian cancer, metabolomics, proteomics, circulating tumor DNA

## Abstract

Epithelial ovarian cancer (EOC) is diagnosed at an advanced stage in over half of the patients and its prognosis remains unfavorable. CA125, one of the most frequent positive tumor markers in patients with EOC, has certain limitations. Therefore, more accurate clinical biomarkers are needed. Liquid biopsy with cancer related molecules, such as circulating tumor DNA, is a new option for cancer diagnosis and prognosis. We explored the potential of plasma metabolomic and proteomic analyses as novel monitoring methods for the patients with EOC. Of seven patients, six experienced disease recurrence or progression. CA125 plasma measurements were conducted for disease monitoring. Plasma metabolome and proteome analyses were performed using liquid chromatography–tandem mass spectrometry. Ten and four metabolome indicators were significantly increased and decreased in association with chemotherapeutic resistance, respectively. In addition, thirty-seven and nine proteins displayed high and low levels associated with chemotherapeutic resistance, respectively. Several metabolome pathways and protein concentrations corresponded to the clinical course of each patient. This pilot study suggested the potential of the assessment of metabolome and proteome analysis as a useful tool for developing novel monitoring biomarkers for patients with recurrent EOC.

## 1. Introduction

More than half of patients with epithelial ovarian cancer (EOC) are diagnosed at the advanced stage, with 70% of advanced (stage III/IV) EOC relapsing within 3 years [1]. Recent international phase III trials have shown that maintenance therapy with bevacizumab and/or PARP inhibitors improves progression-free survival in patients with advanced EOC who have responded to initial chemotherapy, but disease recurrence is still inevitable, and a cure is difficult to achieve in many patients with advanced EOC [2,3,4,5,6].

CA125 is one of the tumor markers with a high positivity rate in patients with EOC, with 80–85% positivity when a cut-off value of 35 U/mL is used [7,8]. Regarding disease recurrence, more than 80% of patients with recurrent EOC are positive for a doubling of CA125 from the upper limit [9]. Both the National Institutes of Health consensus statement and European Society of Medical Oncology consensus conference recommend the measurement of CA125 levels, as well as medical consultation and physical examinations as a routine follow-up after initial treatment for EOC [1,10]. In addition, CA125 is universally used as a marker for monitoring therapeutic response [11,12,13]. However, CA125 can be affected by benign diseases, such as endometriosis and inflammation, and sensitivity and specificity of CA125 are not high enough for it to be used as a single clinical biomarker [14]. There is a need for the establishment of more reliable clinical biomarkers.

With the advances in omics technologies, liquid biopsies have attracted attention as a new option for cancer diagnosis and monitoring [15,16]. Particularly, emerging evidence supports the potential of monitoring tumor DNA circulating in the peripheral blood (ctDNA) as a novel clinical biomarker for various carcinomas [17,18,19]. Importantly, ctDNA and a variety of cancer-associated molecules, including metabolites and proteins, are notable targets for liquid biopsy in oncology [20,21,22].

In contrast to nucleic acid analyses, metabolomics and proteomics require mass spectrometry. Applying a comprehensive analysis of circulating cancer-associated proteins or metabolites to clinical biomarkers has long been challenging owing to the difficulty in standardizing analytic processes, including sample preparation, column selection, and quantification methods [21,23,24]. Recently, we demonstrated the significant difference in plasma metabolomic profile between patients with major gynecologic cancer and healthy controls using the MxP^®^ Quant 500 kit, a liquid chromatography–tandem mass spectrometry (UHPLC-MS/MS)-based product, which quantifies more than 500 metabolites with good reproducibility [25,26,27,28]. In addition, advances in proteomics have enabled researchers to perform analyses with high accuracy and reproducibility [29,30].

To overcome the current limitations in the therapeutic monitoring using CA125 measurements for patients with EOC, this pilot study was launched to identify novel monitoring strategies for EOC by analyzing sequentially collected plasma samples from seven patients using metabolome and proteome analyses. In this study, each plasma sample was classified into two groups according to treatment response, and the metabolomic and proteomic profiles were compared to evaluate the association between plasma metabolites and proteins and clinical response.

## 2. Results

### 2.1. Patient Characteristics and Clinical Course

Seven patients were enrolled in the study. Patient characteristics are summarized in Table 1. During the study period, six of the seven patients experienced disease recurrence or progression. No recurrence was observed in one patient who was treated with niraparib maintenance therapy after confirmation of a clinical complete response with the primary treatment. The clinical course and transition of CA125 and ctDNA in each patient are summarized in Figure S1. Information on the probes used to detect ctDNA in each patient is summarized in Table S1. Somatic mutations detected in the seven patients were also summarized in Table S2.

Each of the seven patients had multiple blood samplings, and plasma samples at each time point were classified into two groups: good-response and poor-response. The number of samples in the good- and poor-response groups for each patient is shown in Table 2.

### 2.2. Data Processing for Metabolome and Proteome Analysis

**Figure 1:**
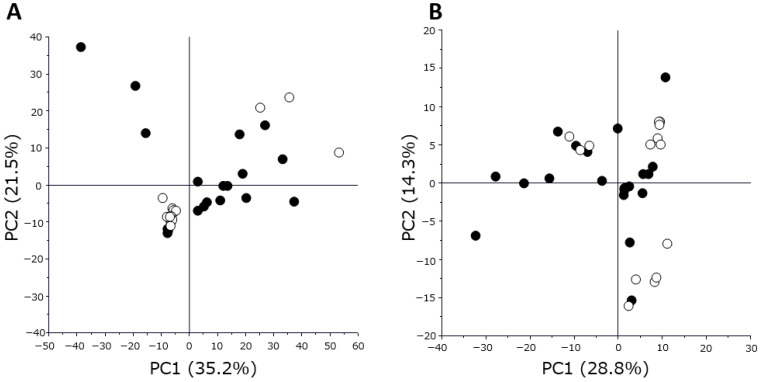
Statistical analysis of good- and poor-response groups using metabolome and proteome data. (**A**) PCA score plot of metabolome data. (**B**) PCA score plot of proteome data. White and black circles indicate the good- and poor-response groups, respectively.

The 222 metabolism indicators and 399 proteins were selected on the basis of <20% of missing values for the statistical analysis. In addition, 208 metabolic indices and 399 proteins were selected for volcano plot analysis because they contained at least three significant values in each group. The ratio of metabolome indicators to protein levels relative to the time of blood sampling prior to cancer resection was statistically analyzed, as the results of the principal competent analysis (PCA) indicated that metabolomic and proteomic data varied widely among individuals (Figure 1).

### 2.3. Comparison of Metabolism Indicators Between Poor Good Response to Chemotherapy

**Figure 2:**
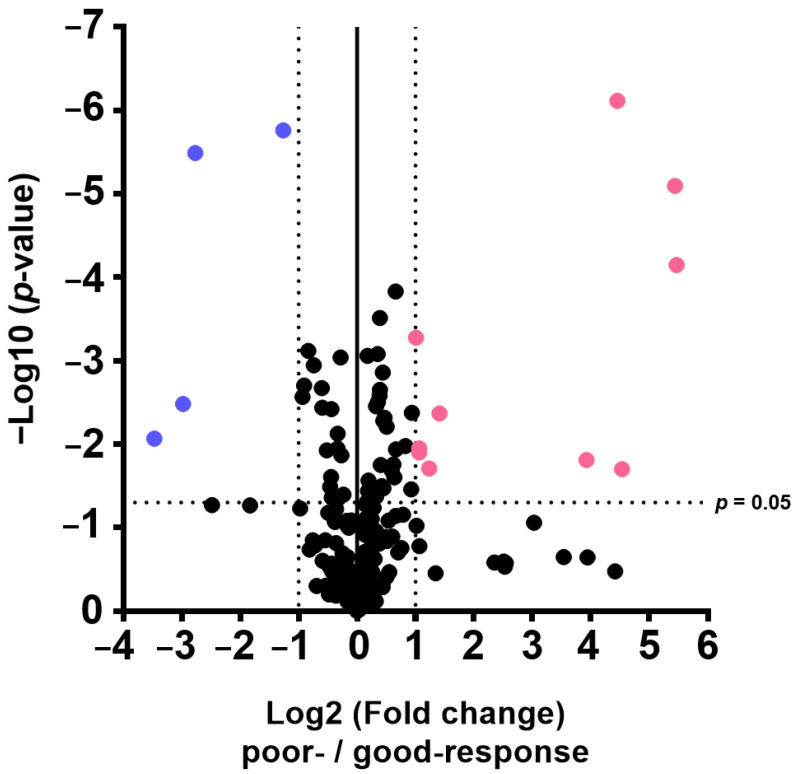
Volcano plot analysis using metabolite indicators. FDR-adjusted *p* < 0.05, log2 fold change > 1 or <−1 was considered significant. The pink and blue plots show the indexes that increased and decreased in the poor-response group, respectively.

**Figure 3:**
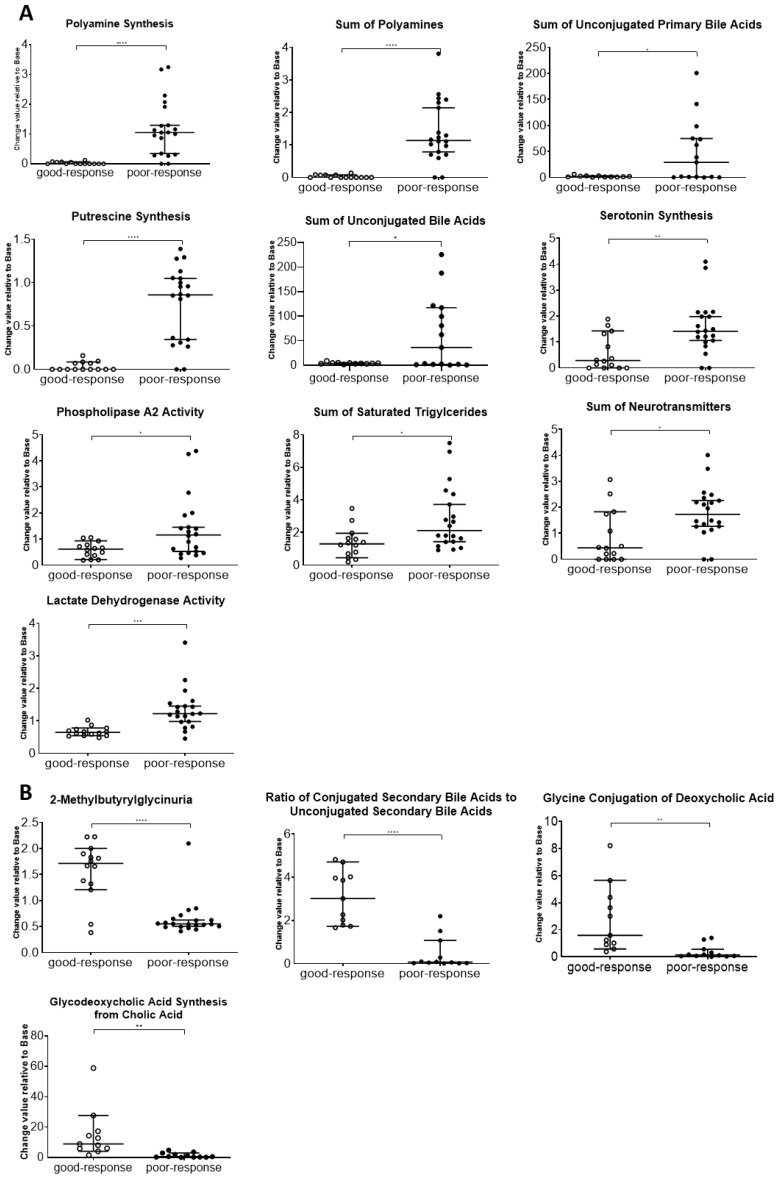
Box plots with individual data points for metabolite indicators in the good- and poor-response groups. The 14 indicators that showed significant differences in the volcano plot analysis are shown. (**A**) Metabolite indicators significantly increased in the poor-response group. (**B**) Metabolite indicators significantly decreased in the poor-response group. * *p* < 0.05, ** *p* < 0.01, *** *p* < 0.005, and **** *p* < 0.001.

Fourteen metabolism indicators were detected in the two groups (poor and good response to chemotherapy, FDR-adjusted *p* < 0.05, log2 fold change > 1 or <−1; Table 3). Ten metabolism indicators (polyamine synthesis, sum of polyamines, sum of unconjugated primary bile acids, putrescine synthesis, sum of unconjugated bile acids, serotonin synthesis, phospholipase A2 (PLA2) activity, sum of saturated triglycerides, sum of neurotransmitters, and lactate dehydrogenase activity) were displayed at a high level, and four metabolism indicators (2-methylbutyrylglycinuria, ratio of conjugated secondary bile acids to unconjugated secondary bile acids, glycine conjugation of deoxycholic acid, and glycodeoxycholic acid synthesis from cholic acid) were indicated at a low level in poor-response samples (Figure 2). Box plots with individual data points for these indicators are also shown in Figure 3.

### 2.4. Comparison of Protein Profiles Between Poor and Good Response to Chemotherapy

**Figure 4:**
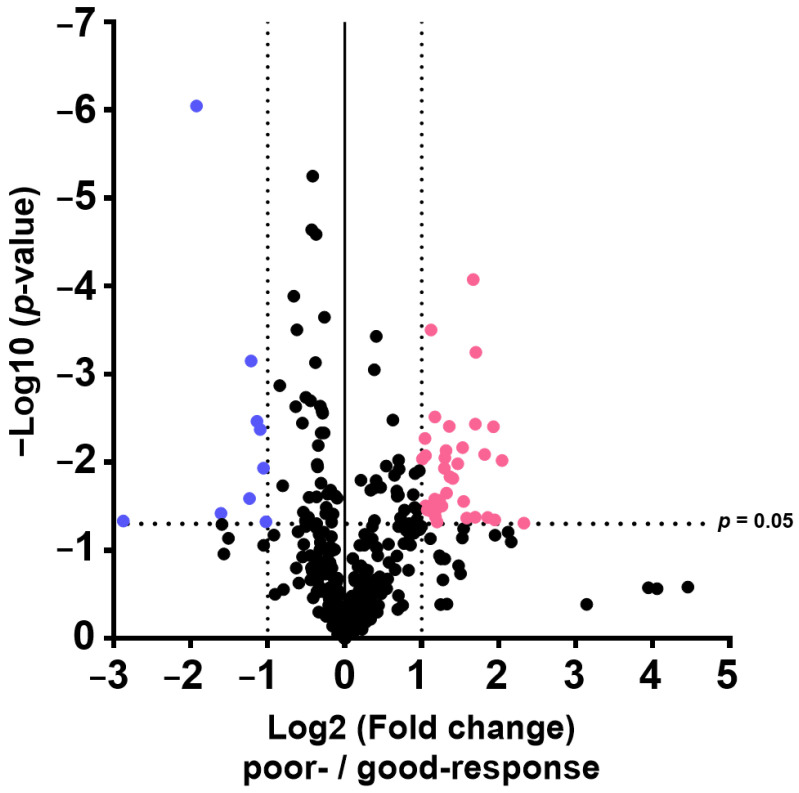
Volcano plot analysis using quantitative protein values. FDR-adjusted *p* < 0.05, log2 fold change > 1 or <−1 was considered significant. The pink and blue plots show the indexes that increased and decreased in the poor-response group, respectively.

**Figure 5:**
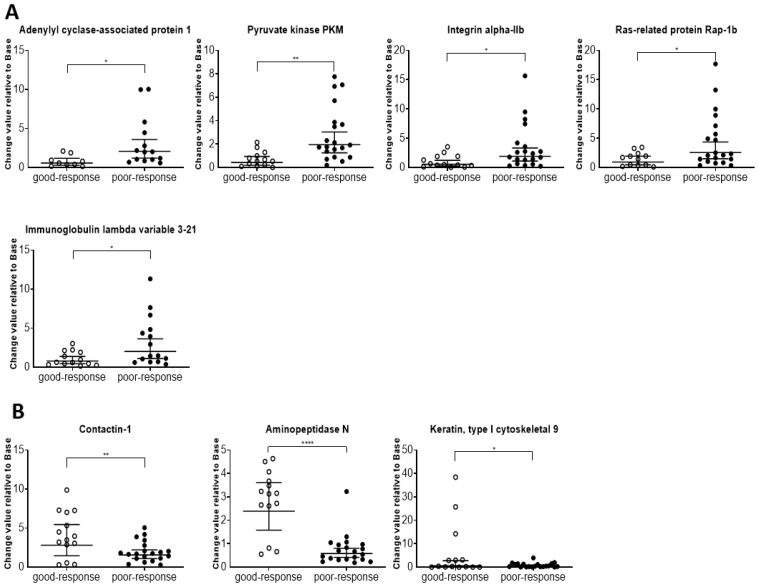
Box plots with individual data points for proteins in the good- and poor-response groups. Eight proteins that meet the following conditions are shown: VIP score > 2, log2 fold change > 1 or <−1, FDR-adjusted *p* < 0.05. (**A**) Proteins with significantly increased amounts in the poor-response group. (**B**) Proteins with significantly decreased amounts in the poor-response group. * *p* < 0.05, ** *p* < 0.01, and **** *p* < 0.001.

Forty-six proteins were detected in the two groups (poor and good response to chemotherapy, FDR-adjusted *p* < 0.05, log2 fold change > 1 or <−1; Table 4). Thirty-seven proteins displayed high levels, and nine (regulator of telomere elongation helicase 1, putative trypsin-6, contactin-1, exostosin-like 2, adhesion G-protein coupled receptor G6, immunoglobulin heavy variable 3-7, cathepsin D, aminopeptidase N, and keratin, and type I cytoskeletal 9) showed low levels in poor-response samples (Figure 4). The eight proteins that satisfy the conditions VIP score > 2, log2 fold change > 1 or <−1, and FDR-adjusted *p*-value < 0.05 are shown in Figure 5.

The pathways identified in the enrichment analysis of the 37 proteins at high level in the poor-response group were cancer-associated, including high-kinase activity BRAF mutant, RAF1 mutant, paradoxical activation of RAF by kinase-inactive BRAF, downstream RAS mutant, moderate kinase activity BRAF mutant, RAS mutants, BRAF and RAF1 fusion, and oncogenic MAPK signaling (Table 5). Integrin beta-3, integrin alpha-IIb, vinculin, actin, cytoplasmic 1, Ras-related protein Rap-1b, and Talin-1 were implicated in these pathways. The enrichment analysis of the nine low-level proteins in the poor-response group showed that the metabolism of angiotensinogen to angiotensins was associated with aminopeptidase N and cathepsin (Table 6).

## 3. Discussion

The current study demonstrated that the profiles of plasma metabolites and proteins were well discriminated based on disease recurrence or the therapeutic response. Together with the results of subsequent analyses for the sequential monitoring of selected metabolite and protein indicators, plasma metabolomic and proteomic analyses may be considered effective strategies for identifying novel clinical biomarkers for monitoring early disease recurrence or therapeutic response in patients with EOC.

The advantage of liquid biopsy is not only accurate disease monitoring but also tailoring therapeutic interventions. Regarding ctDNA in colorectal cancer, some studies, including a phase II randomized clinical study, have indicated the potential benefit of ctDNA as a decision-making marker for the application of adjuvant chemotherapy [31,32]. A phase III trial is also underway to investigate the benefit of adjuvant therapy in patients with stage II–IV radically resectable colorectal cancer presenting positive ctDNA four weeks after surgery [33]. The results of the current study, in combination with that of our previous work on EOC [26], indicate that plasma metabolomic and/or proteomic analysis may also be useful for personalizing therapeutic strategies for patients with EOC.

Another potential benefit of liquid biopsy is early intervention at the time of disease recurrence. A randomized controlled trial (MRC OV5/EORTC55955 study) examining the benefit of early therapeutic intervention based solely on CA125 elevation without any evidence of disease on clinical imaging did not demonstrate an advantage of early intervention in patient prognosis [34]. As mentioned in the introduction, the sensitivity and specificity of CA125 are not sufficiently high to accurately detect disease recurrence. Metabolomic and/or proteomic analysis, ctDNA liquid biopsy, or a combination of these may enable clinicians to detect at or before disease recurrence more precisely than CA125. Recently, molecular targeted therapies, such as anti-angiogenic agents and PARP inhibitors, have been implemented in clinical practice for patients with advanced or recurrent EOC, and the development of antibody-drug conjugates is being accelerated. Despite the discouraging outcome of the MRC OV5/EORTC55955 study, patients with advanced or recurrent EOC now have more treatment options. Therefore, if it becomes possible to diagnose recurrence earlier and more accurately using, for example, metabolomic and/or proteomic analysis, early intervention for recurrent EOC may contribute to extending the overall survival of advanced EOC; then, it may be worth re-evaluating the significance of early treatment intervention based on extremely early diagnosis of recurrence.

The results of this study also indicate that plasma metabolome and proteome analysis would help reveal the mechanisms of chemoresistance or identify vulnerabilities of recurrent EOC. We demonstrated that the 14 metabolite and 46 protein indicators were significantly altered in response to chemotherapy in patients with EOC, after excluding highly abundant proteins using the MxP^®^ Quant 500 and iST-BCT kits combined with abundant protein depletion columns.

Metabolomic reprogramming in cancer cells, including enhancement of glycolysis in ovarian cancer cells, is well known. Enhanced glycolysis in cancer cells is known as the Warburg effect, discovered in the 1920s, and is considered beneficial for proliferation, invasion, metastasis, and chemotherapy resistance by providing by-products from the glycolytic pathway [35,36]. Consistently, our study indicated that the activities of phosphoglycerate kinase 1, pyruvate kinase PKM, glyceraldehyde-3-phosphate dehydrogenase, alpha-enolase, and lactate dehydrogenase were higher in the poor-response group than in the good-response group. In addition, the facilitation of polyamine synthesis in cancer cells is known as metabolic reprogramming. High levels of polyamines in tumor cells have been reported to be caused by dysregulated metabolism and increased uptake from the extracellular environment [37,38,39,40,41]. The upregulation of MYC expression observed in many cancer cells induces ornithine decarboxylase expression, which enhances polyamine synthesis [38,42,43]. Our results also showed high levels of polyamines, including putrescin, which is consistent with previous reports. Thus, these results suggest that the enhancement of glycolysis and elevated polyamine levels in the plasma reflect the rapid proliferation of cancer cells, indicating poor response to chemotherapy. Serotonin, a neurotransmitter, is mitogenic in ovarian cancer [44]. Nocito et al. reported that angiogenesis is induced by a decrease in the production of circulating angiostatin caused by the reduction in matrix metallopeptidase 12 (MMP-12) expression in tumor-associated macrophages at the early stages of tumor development [45]. Furthermore, peripheral serotonin eventually leads to cancer cell growth by suppressing the growth and function of tumor-associated CD8^+^ T cells and promoting PD-L1 expression in tumor cells in the tumor microenvironment [46]. Growth of ovarian cancer cell lines was suppressed by treatment with a serotonin antagonist [47]. In line with all these findings, our results suggest that high plasma serotonin levels induce cancer cell proliferation and indicate a poor response to chemotherapy.

Triglycerides, the principal energy source in the human body, are reserved in adipocytes and peripheral tissues [48]. We previously indicated that the level of triglycerides in the plasma of patients with EOC was higher than that in healthy controls [26]. Additionally, the uncontrolled proliferation of cancer cells necessitates a substantial quantity of lipids to form the cell membranes and organelles of daughter cells [49]. Therefore, in poor-response samples, higher levels of saturated triglycerides compared with those in good-response samples were considered to support the findings of previous studies. For acylcarnitines known to be related to fatty acid metabolism, the sum of monosaturated fatty acid acylcarnitine and the ratio of valerylcarnitine to propionylcarnitine were significantly different between the poor- and good-responders in this study. These changes in the ratio of carnitines resulted in low levels of valerylcarnitine and high levels of propionylcarnitine in the poor-response cases. Huang et al. reported a higher propionylcarnitine level in plasma of patients with ovarian cancer than that in healthy controls [50]. Additionally, carnitine palmitoyltransferase 1A (CPT1A), which is a rate-limiting enzyme of fatty acid β-oxidation, was upregulated in ovarian cancer cells [51]. Thus, this alteration of carnitine species in the poor-responders group is likely a part of lipid remodeling in ovarian cancer cells.

PLA2 activity, which is an indicator of the hydrolysis of phosphatidylcholine (PC) aa C36:5 into lysophosphatidylcholine (LPC) a C16:1 and arachidonic acid, a polyunsaturated omega-6 fatty acid 20:4, was higher in the poor-response than that in good-response cases. In a previous study, PLA2 activity was upregulated in ovarian cancer compared with that in normal and benign tissues [52], and high and low levels of LPCs and PCs, respectively, were reported by Zhang et al. [53]. Our data also showed lower PC aa C36:5 and higher LPC a C16:1 levels. Additionally, arachidonic acid produced through PLA2 activity, which promotes the occurrence and development of tumors [54], has been reported to be higher in the plasma of patients with ovarian cancer than that in controls [55]. This suggests that increased PLA2 activity reflects tumor progression.

Our results showed that unconjugated bile acid levels were high in the plasma of patients with poor response. Unconjugated bile acids are products of primary bile acids produced by the gut microbiota [56]. Dysbiosis, known as oncobiosis, has been observed in the gut microbiota of patients with ovarian cancer, which induces resistance to chemotherapy [57,58]. In addition, the enterohepatic circulation of bile acids changes and resolution of bile acids is inhibited in patients with ovarian cancer [59]. Thus, this alteration in unconjugated bile acids indicates oncobiosis caused by ovarian cancer.

Regulators of telomere elongation helicase 1 (RTEL1) are of high interest because RTEL1 is related to the telomere region, which contributes to cancer cell proliferation. RTEL1 induces both deficiency and overexpression, leading to instability of the genome and triggering oncogenic transformations [60]. The metabolism of angiotensinogen to angiotensins found in the enrichment analysis involved nine low-level proteins that were considered to be related to cachexia caused by cancer [61]. Additionally, cancer disease pathways showed 37 proteins at high levels, including integrin beta-3, integrin alpha-IIb, vinculin, actin, cytoplasmic 1, Ras-related protein Rap-1b, and talin-1. Thus, these protein profiles seem to indicate the patient’s condition and have potential applications in chemotherapy management.

The sample size was a major limitation of the current study from the perspective of clinical application. It should also be noted that the transition of neither CA125, ctDNA, proteins, nor metabolomic indicators measured in this study perfectly corresponded to the clinical course of the seven patients, suggesting the importance of investigating combination markers for a more reliable liquid biopsy. In the future, it is also expected that the alterations in metabolites and proteins in the plasma shown in the present study will be verified in an in vitro experimental system. Based on the results of this pilot study, we integrated candidate profiles from metabolic and proteomic analyses, and were able to find the possibility of establishing a combination of highly sensitive and specific clinical monitoring markers for the early diagnosis of recurrence and the construction of disease monitoring markers for predicting treatment efficacy of EOC. To optimize and verify the plasma metabolic and proteomic profiles related to early diagnosis of recurrence and prediction of treatment efficacy, it is necessary to introduce innovative new technologies such as artificial intelligence and to strategically build up a series of step-by-step verifications.

## 4. Materials and Methods

### 4.1. Ethical Considerations

This study was approved by the internal review board of the Tohoku University School of Medicine (approval number: 2021-1-607).

### 4.2. Patient Enrollment

Patients who were being treated for recurrent disease, undergoing maintenance therapy, or on routine follow-up after primary or recurrent treatment at Tohoku University Hospital (Sendai, Japan) with a diagnosis of primary ovarian, fallopian tube, or peritoneal carcinoma were eligible for this study. The patients were enrolled between December 2021 and March 2022. After obtaining written informed consent, plasma was collected from patients at each visit or on day 1 of each chemotherapy cycle until the end of March 2023. Plasma was stored in the Tohoku University Clinical Biobank (TUCB) until metabolomic and proteomic analyses or liquid biopsy of ctDNA.

### 4.3. Clinical Information

Clinical information was extracted from clinical records and databases, including age at enrollment, International Federation of Gynecology and Obstetrics 2014 stage, histological subtype, and treatment history before enrollment [62]. Serum CA125 levels, treatment context, tumor response to treatment, and patient outcomes were monitored during the study period. Tumor response was evaluated in accordance with the criteria defined by Response Evaluation Criteria in Solid Tumors Version 1.1.

### 4.4. Chemical Reagents

The following reagents were purchased: pyridine (TCI, Tokyo, Japan), ethanol (Nacalai Tesque, Kyoto, Japan), methanol (Kanto Kagaku, Tokyo, Japan), phenyl isothiocyanate, formic acid, ammonium acetate (all from Wako Pure Chemical Industries, Osaka, Japan), and ammonium bicarbonate (Cell Science & Technology Institute, Inc., Miyagi, Japan), all of which were of the highest quality commercially available. Pooled healthy human plasma was purchased from Innovative Research (Lot No. 26393, Innovative Research, Novi, MI, USA) and aliquoted as the global quality control plasma sample.

### 4.5. Genomic Sequencing

DNA was extracted from the primary tumor specimen and blood cells of each patient were stored at TUCB. DNA was subjected to whole-exome sequencing (WES) or whole-genome sequencing (WGS) to identify somatic variants. WES was performed at the Tohoku University Advanced Research Center for Innovations in Next-Generation Medicine. WGS was obtained from Haplo Pharma, Inc. (Sendai, Japan). The sequencing data were mapped using bwa-mem 0.1.17. Somatic variant calls were made using the GATK Best Practice Workflow for Somatic short variant discovery. Pathogenic genomic variants in the tumors of each patient were determined using ClinVar, ToMMo 38KJPN, and snpEff [63,64,65].

### 4.6. ctDNA Measurement

ctDNA was quantified as previously described [17]. Briefly, the ctDNA was extracted from 10 mL of blood stored in PAX gene Blood ccf tubes (QIAGEN, Hilden, Germany) using the QIAamp Circulating Nucleic Acid Kit (QIAGEN). Based on the results of somatic gene alteration, the corresponding BIORAD Droplet digital PCR (ddPCR) mutation assay primers and probes were prepared for each patient. The ddPCR was performed using a QX200 AutoDG Droplet Digital PCR IVD System (Bio-Rad, Hercules, CA, USA) according to the manufacturer’s instructions. DNA extracted from primary tumor specimens was used as positive control in each case.

### 4.7. Sample Preparation for Metabolomics

Wide target metabolomics was performed using a MxP^®^ Quant 500 kit (Biocrates Life Science AG, Innsbruck, Austria) with an ultra-performance liquid chromatograph (ACQUITY UPLC I-Class, Waters Corporation, Milford, MA, USA) connected to a triple quadrupole mass spectrometer (MS; Xevo TQ-XS, Waters Corporation) as previously described [66,67,68]. The blank solution (10 μL), calibration standard solutions, quality control solutions, and plasma samples were prepared and measured according to the MxP^®^ Quant 500 kit manual. Metabolite concentrations were calculated from the exported raw files using MetIDQ^TM^ version Oxygen software (Biocrates Life Science AG).

### 4.8. Sample Preparation for Proteomics

Proteomics was performed using a previously reported method, with several modifications [69]. To remove the high-abundance proteins in the plasma samples, 10 μL of plasma sample was treated with the Pierce^®^ Top 14 Abundant Protein Depletion Spin Columns Kit (Thermo Fisher Scientific, Waltham, MA, USA), and then the protein concentration was determined with a Pierce^®^ BCA Protein Assay kit (Thermo Fisher Scientific) according to the manufacturer’s instructions. The plasma proteins obtained from the Top 14 kit were digested in solution using an iST-BCT kit (PreOmics GmbH, Planegg-Martinsried, Germany). Plasma protein (30 μg) was transferred to another tube redissolved in LYSE buffer after being vacuum-dried. To cleave disulfide bonds, reduction and alkylation were carried out for 10 min at 95 °C. The reduced and alkylated protein solutions were enzymatically digested by adding 50 μL of DIGEST solution for 3 h at 37 °C. After digestion, the sample peptides were purified using a cartridge and two types of WASH solutions. Purified peptides were vacuum-dried and resuspended in 60 μL of LOAD solution to reach a final protein concentration of 0.5 μg/μL.

The sample solutions were analyzed under the following instrument conditions: liquid chromatography–mass spectrometry analysis was performed using an EASY-nLC 1000 UPLC system (Thermo Fisher Scientific) and Orbitrap Fusion^TM^ Tribrid^TM^ mass spectrometer (Thermo Fisher Scientific). The peptide sample (2 µL) was loaded in eluent A (0.1% FA) into a trap column (C18, 3 μm particle, 2 cm length, 75 μm ID, Acclaim PepMap 100; Thermo Fisher Scientific) and separated using a reverse-phase analytical column (Nano HPLC CAPILLARY COLUMN, 75 μm × 12.5 cm, 3 μm, ODS; Nikkyo Technos, Co., Ltd., Tokyo, Japan) on a Nanospray Flex Ion Source system (Thermo Fisher Scientific). The 1 μg of peptide was separated using a gradient program for 60 min with eluents A and B (0.1% FA in 80% acetonitrile) at a constant flow rate of 300 nL/min. The gradient program was set as follows: an initial elution with 5% B for 1 min, a gradient to 40% B for 61 min, a climbing period of 95% for 63 min, and a holding period of 95% for 80 min. To detect peptides ionized by electrospray voltage at 2.0 kV, primary MS was performed using an Orbitrap at 60,000 mass resolution in the scan range of 375–1600 *m*/*z*. The automatic gain control (AGC) was set to 4 × 10^6^, and the maximum injection time (MIT) was set to 50 ms. The maximum intensity threshold was set to 1 × 10^20^ and the dynamic exclusion duration was set to 20 s to prevent the precursor ions from being repeatedly scanned. Data were collected in data-dependent acquisition mode. Secondary MS was performed using a quadrupole at a 0.7 isolation window. Activation was induced using collision dissociation at 35% collision energy for 10 ms in the fixed collision energy mode. The detector used an ion trap with an MIT of 35 ms and a target AGC value of 1 × 10^5^. The third MS was conducted using a quadrupole with an isolation window of 0.7 and HCD activation at a 55% collision energy in the fixed collision energy mode. The detector used an Orbitrap at 50,000 mass resolution in the scanning range of 100–500 *m*/*z*. The AGC was set to 15 × 10^4^, and the MIT was set to 86 ms.

### 4.9. Statistical Analysis

Proteome and metabolome data analyses were performed using GraphPad Prism 8 software (GraphPad Software Inc., San Diego, CA, USA) for volcano plot analysis and boxplot drawing. The PCA, orthogonal partial least squares discriminant analysis, and variable importance for projection were performed using SIMCA software Version 13.0.3 (Sartorius, San Francisco, CA, USA). *p*-values were calculated using Wilcoxon rank sum test. Differences with *p* < 0.05 were considered statistically significant.

## 5. Conclusions

This pilot study clarified the potential of the assessment of metabolic and proteomic analysis as a useful tool for developing novel monitoring biomarkers for patients with recurrent EOC. Further studies are warranted to apply these techniques for the detection of early disease recurrence or the prediction of efficacy of therapeutic interventions in clinical practice.

## Figures and Tables

**Table 1:** Main characteristics of patients with epithelial ovarian cancer enrolled in this study.

		Range (Median)
Total (*n*)		7
Age		63–65 (67)
		Number of patients
Diagnosis		
	Ovarian cancer	4
	Fallopian tube cancer	2
	Peritoneal cancer	1
Histology		
	High-grade serous carcinoma	5
	Clear cell carcinoma	1
	Mucinous carcinoma	1
Disease recurrence/progression during the study period		
	Yes	6
	No	1

**Table 2:** Number of points for good- and poor-response groups per patient.

Case No.	Good-Response	Poor-Response
1	3	5
2	5	1
3	0	3
4	0	3
5	0	1
6	0	7
7 (No recurrence)	6	0
Total	14	20

**Table 3:** Metabolism indicators detected based on FDR-adjusted *p* < 0.05, log2 fold change > 1 or <−1.

Metabolism Indicator	Log2 Fold Change	FDR
Polyamine Synthesis	5.47	2.00 × 10^−4^
Sum of Polyamines	5.44	2.81 × 10^−5^
Sum of Unconjugated Primary Bile Acids *	4.53	1.98 × 10^−2^
Putrescine Synthesis	4.45	1.08 × 10^−5^
Sum of Unconjugated Bile Acids *	3.92	1.80 × 10^−2^
Serotonin Synthesis	1.41	7.47 × 10^−3^
Phospholipase A2 Activity	1.23	2.10 × 10^−2^
Sum of Saturated Trigylcerides	1.06	1.59 × 10^−2^
Sum of Neurotransmitters	1.06	1.57 × 10^−2^
Lactate Dehydrogenase Activity	1.01	1.23 × 10^−3^
2-Methylbutyrylglycinuria	−1.25	1.22 × 10^−5^
Ratio of Conjugated Secondary Bile Acids to Unconjugated Secondary Bile Acids	−2.74	1.51 × 10^−5^
Glycine Conjugation of Deoxycholic Acid	−2.94	6.56 × 10^−3^
Glycodeoxycholic Acid Synthesis from Cholic Acid *	−3.47	1.33 × 10^−2^

*: VIP score > 2.

**Table 4:** Proteins detected based on FDR-adjusted *p* < 0.05, log2 fold change > 1 or <−1.

Accession	Description	Log2 Fold Change	FDR
Q14766	Latent-transforming growth factor beta-binding protein 1	2.33	4.89 × 10^−2^
P00558	Phosphoglycerate kinase 1	2.05	2.31 × 10^−2^
Q01518	Adenylyl cyclase-associated protein 1 *	1.95	4.92 × 10^−2^
P14618	Pyruvate kinase PKM *	1.93	1.81 × 10^−2^
P06702	Protein S100-A9	1.86	4.85 × 10^−2^
P50552	Vasodilator-stimulated phosphoprotein	1.82	2.50 × 10^−2^
P08567	Pleckstrin	1.70	6.46 × 10^−3^
P04406	Glyceraldehyde-3-phosphate dehydrogenase	1.70	2.11 × 10^−2^
P08514	Integrin alpha-IIb *	1.69	4.95 × 10^−2^
Q05682	Caldesmon	1.67	1.93 × 10^−3^
P61224	Ras-related protein Rap-1b *	1.58	4.80 × 10^−2^
P80748	Immunoglobulin lambda variable 3–21 *	1.54	4.41 × 10^−2^
Q13201	Multimerin-1	1.53	2.40 × 10^−2^
P07996	Thrombospondin-1	1.47	2.39 × 10^−2^
Q9Y490	Talin-1	1.41	2.91 × 10^−2^
O95810	Caveolae-associated protein 2	1.37	2.92 × 10^−2^
P04040	Catalase	1.36	2.00 × 10^−2^
P06753-2	Isoform 2 of Tropomyosin alpha-3 chain	1.32	4.12 × 10^−2^
P05106	Integrin beta-3	1.32	2.41 × 10^−2^
Q15166	Serum paraoxonase/lactonase 3	1.30	2.40 × 10^−2^
P02775	Platelet basic protein	1.29	2.45 × 10^−2^
P21333	Filamin-A	1.26	4.50 × 10^−2^
P09493-8	Isoform 8 of Tropomyosin alpha-1 chain	1.23	4.44 × 10^−2^
P05109	Protein S100-A8	1.20	4.86 × 10^−2^
P35579	Myosin-9	1.19	4.66 × 10^−2^
P07737	Profilin-1	1.19	4.65 × 10^−2^
P06733	Alpha-enolase	1.18	5.04 × 10^−2^
P18206	Vinculin	1.18	4.46 × 10^−2^
Q15485	Ficolin-2	1.17	2.33 × 10^−2^
P16671	Platelet glycoprotein 4	1.17	4.45 × 10^−2^
P10599	Thioredoxin	1.12	4.81 × 10^−3^
P12814	Alpha-actinin-1	1.11	4.54 × 10^−2^
P20742	Pregnancy zone protein	1.07	4.56 × 10^−2^
O14950	Myosin regulatory light chain 12B	1.05	4.61 × 10^−2^
P48059	LIM and senescent cell antigen-like-containing domain protein 1	1.05	2.40 × 10^−2^
P02786	Transferrin receptor protein 1	1.04	2.05 × 10^−2^
P60709	Actin, cytoplasmic 1	1.01	2.33 × 10^−2^
Q9NZ71	Regulator of telomere elongation helicase 1	−1.03	4.93 × 10^−2^
Q8NHM4	Putative trypsin-6	−1.06	2.54 × 10^−2^
Q12860	Contactin-1 *	−1.09	1.76 × 10^−2^
Q9UBQ6	Exostosin-like 2	−1.15	2.24 × 10^−2^
Q86SQ4	Adhesion G-protein coupled receptor G6	−1.22	6.49 × 10^−2^
P01780	Immunoglobulin heavy variable 3-7	−1.25	4.55 × 10^−2^
P07339	Cathepsin D	−1.60	4.71 × 10^−2^
P15144	Aminopeptidase N *	−1.94	4.12 × 10^−5^
P35527	Keratin, type I cytoskeletal 9 *	−2.84	4.95 × 10^−2^

*: VIP score > 2.

**Table 5:** Enrichment analysis of the proteins at high level in the poor-response group using the reactome pathway tool (FDR-adjusted *p*-value < 0.001).

Reactome Pathway ID	Pathway Name	Entities *p*-Value	Entities FDR
R-HSA-114608	Platelet degranulation	2.22 × 10^−16^	4.90 × 10^−14^
R-HSA-76005	Response to elevated platelet cytosolic Ca^2+^	3.33 × 10^−16^	4.90 × 10^−14^
R-HSA-76002	Platelet activation, signaling and aggregation	7.13 × 10^−14^	6.99 × 10^−12^
R-HSA-109582	Hemostasis	3.98 × 10^−10^	2.90 × 10^−8^
R-HSA-6802948	Signaling by high-kinase activity BRAF mutants	2.80 × 10^−9^	1.65 × 10^−7^
R-HSA-446353	Cell-extracellular matrix interactions	4.50 × 10^−9^	2.13 × 10^−7^
R-HSA-5674135	MAP2K and MAPK activation	5.13 × 10^−9^	2.13 × 10^−7^
R-HSA-9656223	Signaling by RAF1 mutants	5.91 × 10^−9^	2.13 × 10^−7^
R-HSA-6802955	Paradoxical activation of RAF signaling by kinase inactive BRAF	1.15 × 10^−8^	2.75 × 10^−7^
R-HSA-9649948	Signaling downstream of RAS mutants	1.15 × 10^−8^	2.75 × 10^−7^
R-HSA-6802946	Signaling by moderate kinase activity BRAF mutants	1.15 × 10^−8^	2.75 × 10^−7^
R-HSA-6802949	Signaling by RAS mutants	1.15 × 10^−8^	2.75 × 10^−7^
R-HSA-6802952	Signaling by BRAF and RAF1 fusions	8.40 × 10^−8^	1.85 × 10^−6^
R-HSA-372708	p130Cas linkage to MAPK signaling for integrins	2.05 × 10^−7^	3.49 × 10^−6^
R-HSA-354194	GRB2:SOS provides linkage to MAPK signaling for integrins	2.05 × 10^−7^	3.49 × 10^−6^
R-HSA-6802957	Oncogenic MAPK signaling	3.42 × 10^−7^	5.47 × 10^−6^
R-HSA-354192	Integrin signaling	2.42 × 10^−6^	3.38 × 10^−5^
R-HSA-70171	Glycolysis	6.72 × 10^−6^	9.41 × 10^−5^
R-HSA-76009	Platelet aggregation (plug formation)	9.78 × 10^−6^	1.27 × 10^−4^
R-HSA-70326	Glucose metabolism	1.25 × 10^−5^	1.50 × 10^−4^
R-HSA-445355	Smooth muscle contraction	1.42 × 10^−5^	1.70 × 10^−4^
R-HSA-168249	Innate Immune System	1.56 × 10^−5^	1.71 × 10^−4^
R-HSA-6798695	Neutrophil degranulation	2.02 × 10^−5^	2.22 × 10^−4^
R-HSA-5602498	MyD88 deficiency (TLR2/4)	3.63 × 10^−5^	3.63 × 10^−4^
R-HSA-5603041	IRAK4 deficiency (TLR2/4)	4.22 × 10^−5^	4.03 × 10^−4^
R-HSA-446728	Cell junction organization	4.29 × 10^−5^	4.03 × 10^−4^
R-HSA-195258	RHO GTPase effectors	4.47 × 10^−5^	4.03 × 10^−4^
R-HSA-9636667	Manipulation of host energy metabolism	4.74 × 10^−5^	4.26 × 10^−4^
R-HSA-5627123	RHO GTPases activate PAKs	4.87 × 10^−5^	4.39 × 10^−4^
R-HSA-5686938	Regulation of TLR by endogenous ligand	5.59 × 10^−5^	4.47 × 10^−4^
R-HSA-422475	Axon guidance	6.28 × 10^−5^	5.02 × 10^−4^
R-HSA-9675108	Nervous system development	8.93 × 10^−5^	7.14 × 10^−4^
R-HSA-70263	Gluconeogenesis	9.14 × 10^−5^	7.15 × 10^−4^
R-HSA-3000170	Syndecan interactions	1.02 × 10^−4^	7.15 × 10^−4^

**Table 6:** Enrichment analysis of the proteins at low level in the poor-response group using the reactome pathway tool (FDR-adjusted *p*-value < 0.01).

Reactome Pathway ID	Pathway Name	Entities *p*-Value	Entities FDR
R-HSA-2022377	Metabolism of angiotensinogen to angiotensins	7.38 × 10^−5^	6.4 × 10^−3^

## Data Availability

The data that support the findings of this study are available on request from the corresponding author. The data are not publicly available due to privacy or ethical restrictions. In addition, the data about identifiable human research participants cannot be openly shared.

## Supplementary Materials

The supporting information can be downloaded at https://www.mdpi.com/article/10.3390/ijms26125435/s1.

## Abbreviations

The following abbreviations are used in this manuscript:

AGC	automatic gain control
CT	clinical trial
ctDNA	circulating tumor DNA
DC	docetaxel plus carboplatin
EOC	epithelial ovarian cancer
Gem	gemcitabine
MIT	maximum injection time
MS	mass spectrometry
Nir	niraparib
NOG	nogitecan
NOGBev	nogitecan plus bevacizumab
Ola	olaparib
PCA	principal component analysis
PLDBev	pegylated liposomal doxorubicin plus bevacizumab
TC	paclitaxel plus carboplatin
WES	whole-exome sequencing
WGS	whole-genome sequencing
wTBev	weekly paclitaxel plus bevacizumab
