# Supplementary material for: Sequential Plasma Metabolome and Proteome Analyses to Develop a Novel Monitoring Strategy for Patients with Epithelial Ovarian Cancer: A Pilot Study
Source: Int J Mol Sci. 2025 Jun 6;26(12):5435. doi: 10.3390/ijms26125435 (PMC12192702; doi:10.3390/ijms26125435)
Supplement: Supplementary file 1 [file ijms-26-05435-s001.zip › ijms-3594040-supplementary.pdf]

Supplementary Materials

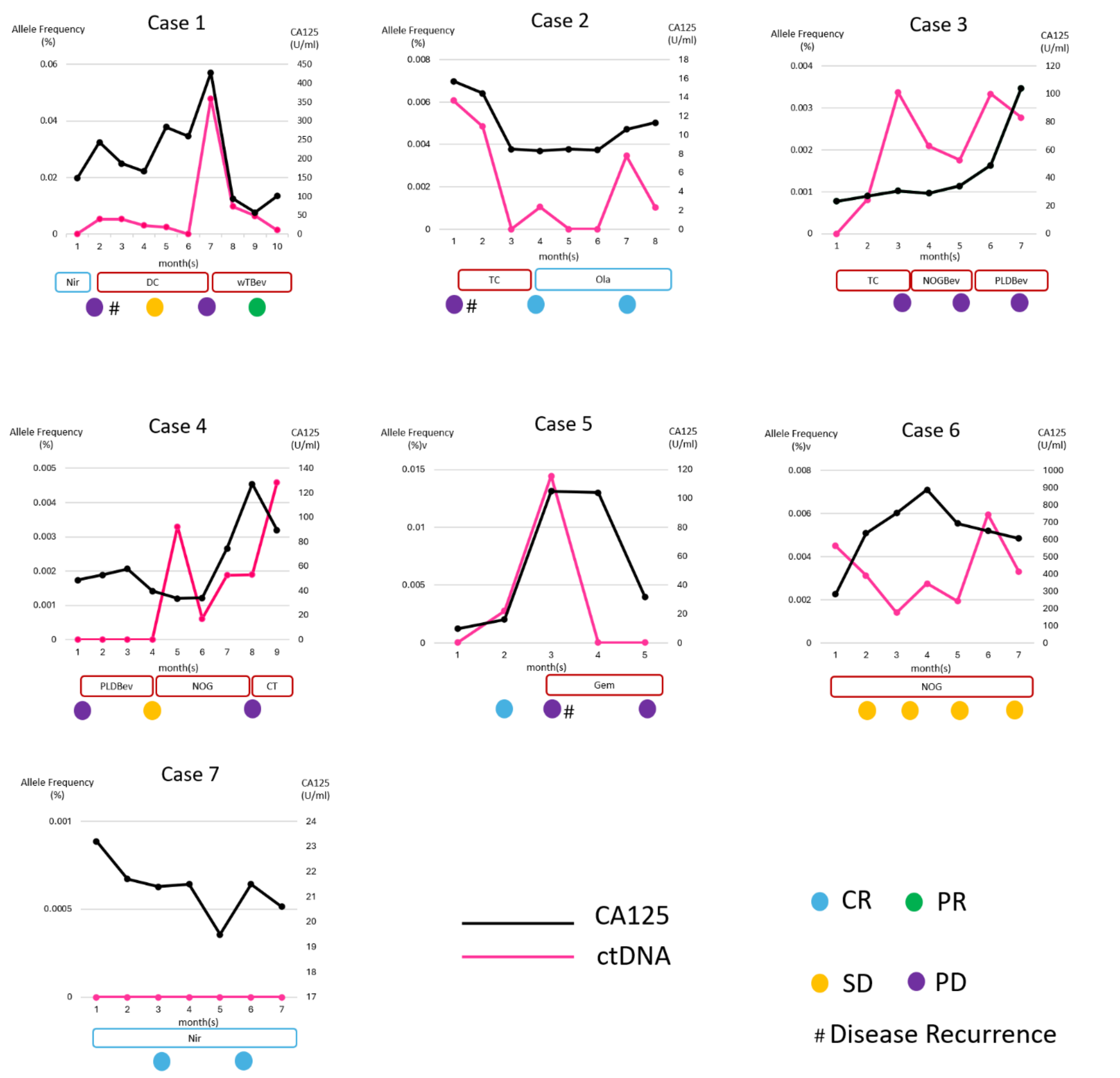

Figure S1. Summary of the clinical course of the transition of CA125 and ctDNA in seven patients. Black and pink lines represent the transitions of CA125 and ctDNA, respectively. The blue, green, yellow, and purple dots represent complete response (CR), partial response (PR), stable disease (SD), and progressive disease (PD), respectively, as determined by clinical imaging.

Table S1. Summary of the probes used for ctDNA detection in the seven patients.

Case No.	Targeted gene alteration		Complementary DNA alteration	BIORAD ddPCR™ mutation assay ID
1	PIK3CA p.His1047Leu	NC_000003.12:g.179234297A>T	c.3140A>T	dHsaMDV2010123
2	TP53 p.Glu349*	NC_000017.11:g.7670664C>A	c.1045G>T	dHsaMDS2514806
3	KRAS p.Gly12Val	NC_000012.12:g.25245350C>A	c.35G>T	dHsaMDV2010005
4	PIK3CA p.Gln546Lys	NC_000003.12:g.179218306C>A	c.1636C>A	dHsaMDS684842111
5	TP53 p.Gly105Val	NC_000017.11:g.7676055C>A	c.314G>T	dHsaMDS600973047
6	TP53 p.Cys176Trp	NC_000017.11:g.7675084G>C	c.528C>G	dHsaMDS2511718
7	TP53 p.Tyr220Cys	NC_000017.11:g.7674872T>C	c.659A>G	dHsaMDS226444183

Table S2. Summary of the somatic mutations found in the seven patients.

Case No.	Gene Symbol	Amino Acid Alteration	Gene Mutation
1	*TP53*	p.His178fs	NC_000017.11:g.7675084del
	PIK3CA	p.His1047Leu	NC_000003.12:g.179234297A>T
	MAP3K1	p.His1058fs	NC_000005.10:g.56882372del
	DDR2	p.Arg752Cys	NC_000001.11:g.162776341C>T
	MC5R	p.Ala228Ala	NC_000018.10:g.13826449G>A
2	*TP53*	p.Glu349*	NC_000017.11:g.7670664C>A
	BRCA1	p.Lys1459*	NC_000017.11:g.43076597T>A
3	*TP53*	p.Phe109Ser	NC_000017.11:g.7676043A>G
	KRAS	p.Gly12Val	NC_000012.12:g.25245350C>A
	ALX3	p.Arg196Trp	NC_000001.11:g.110064595G>A
4	*PIK3CA*	p.Gln546Lys	NC_000003.12:g.179218306C>A
	KRAS	(p.Gly12Ala	NC_000012.12:g.25245350C>G
	NCOR1	p.Gly639fs	NC_000017.11:g.16119424dup
5	*TP53*	p.Gly105Val	NC_000017.11:g.7676055C>A
	GRM7	p.Arg659*	NC_000003.12:g.7578881C>T
	IDS	p.Gln277*	NC_000023.11:g.149496396G>A
6	*TP53*	p.Cys176Trp	NC_000017.11:g.7675084G>C
	KDM5C	p.Leu1381fs	NC_000023.11:g.53193612del
7	*TP53*	p.Tyr220Cys	NC_000017.11:g.7674872T>C
